# Effects of Electrospun Carbon Nanofibers’ Interlayers on High-Performance Lithium–Sulfur Batteries

**DOI:** 10.3390/ma10040376

**Published:** 2017-03-31

**Authors:** Tianji Gao, TrungHieu Le, Ying Yang, Zhihao Yu, Zhenghong Huang, Feiyu Kang

**Affiliations:** 1 Department of Chemical and Environmental Engineering, China University of Mining and Technology, Beijing 100083, China; gmxzxcv@163.com; 2 State Key Laboratory of Power System, Tsinghua University, Beijing 100084, China; lizhongx15@mails.tsinghua.edu.cn; 3 Department of Electrical Engineering, Tsinghua University, Beijing 100084, China; youngharold@qq.com; 4 Laboratory of Advanced Materials, Department of Materials Science and Engineering, Tsinghua University, Beijing 100084, China; zhhuang@mail.tsinghua.edu.cn (Z.H.); fykang@sz.tsinghua.edu.cn (F.K.)

**Keywords:** lithium–sulfur battery, carbon nanofibers interlayer, suppress lithium dendrite, high sulfur loading

## Abstract

Two different interlayers were introduced in lithium–sulfur batteries to improve the cycling stability with sulfur loading as high as 80% of total mass of cathode. Melamine was recommended as a nitrogen-rich (N-rich) amine component to synthesize a modified polyacrylic acid (MPAA). The electrospun MPAA was carbonized into N-rich carbon nanofibers, which were used as cathode interlayers, while carbon nanofibers from PAA without melamine was used as an anode interlayer. At the rate of 0.1 C, the initial discharge capacity with two interlayers was 983 mAh g^−1^, and faded down to 651 mAh g^−1^ after 100 cycles with the coulombic efficiency of 95.4%. At the rate of 1 C, the discharge capacity was kept to 380 mAh g^−1^ after 600 cycles with a coulombic efficiency of 98.8%. It apparently demonstrated that the cathode interlayer is extremely effective at shutting down the migration of polysulfide ions. The anode interlayer induced the lithium ions to form uniform lithium metal deposits confined on the fiber surface and in the bulk to strengthen the cycling stability of the lithium metal anode.

## 1. Introduction

The lithium–sulfur (Li–S) battery is one of the most promising candidates for its high energy density applications, especially in electric vehicles (EVs) due to its low-cost, nontoxic, surprising theoretical specific capacity and energy density of 1675 mAh g^−1^ and 2500 Wh kg^−1^, respectively [1,2]. However, the development of the lithium–sulfur battery for practical applications has been obstructed to date by a series of challenges, including the insulating nature of S_8_, large volume change during the intercalation process and the solubility of polysulfides as intermediate products in liquid electrolytes. Extensive research effort has been made to solve these issues [3,4].

One of the most effective methods is to import an interlayer or modify the separator, which means that the interlayer serves as a barrier to limit soluble polysulfide diffusion and localizes the active material within the cathode side. This in turn facilitates the reutilization of the entrapped active materials in the following cycles, thus improving the capacity retention and cyclic stability of the cells. Nickel foam [5], polypyrrole nanotubes film (PNTF) [6], Fe_3_C/carbon nanofibers (CNF) [7], polypyrrole functional interlayer (PFIL) [8], microporous carbon paper [9,10], carbon paper [11], hollow carbon nanofiber/reduced graphene oxide [12], etc., can be utilized as interlayers in an Li–S battery. However, the interaction between nonpolar carbon and polar lithium polysulfides is not enough to entirely avoid the loss of sulfur [13].

Therefore, in order to promote the interaction between lithium polysulfides and barrier layers, researchers must pay attention to the chemical absorption of polysulfides. The surface modification of porous carbon has been recognized as a useful strategy for constructing carbon materials, since various organic groups can be attached onto its surface. Nitrogen is the most attractive doping element and could substantially improve the carbon wettability, basic property, adsorptive ability, surface polarity, and conductivity [14,15,16,17,18,19,20,21]. Following this concept, nitrogen-doped porous hollow carbon sphere [22], a bifunctional MCNT@PEG [23], and nitrogen/sulfur-codoped graphene sponge [24] have been reported to modify the separator or function as an interlayer. Such complicated and expensive synthesis processes and the low polysulfide adsorption ability of the interlayers are still major barriers to the wide application of the interlayers. However, the rich organic chemistry library provides several reactive intermediates for attacking and modifying carbon frameworks. Such a reaction can limit the carbon loss ratio if a suitable chemical reaction is designed.

Moreover, sulfur loading in the cathode is still inadequate, which greatly offsets the advantage of the high energy density of Li–S batteries. Most sulfur cathodes in the previous investigations had sulfur loads below 2 mg cm^−2^ and a sulfur fraction below 60% [25]. The sulfur fraction in sulfur electrons is well below the average value of 80% in lithium-ion cathodes [26], which implies that it is necessary to develop a cost-effective and straightforward strategy that can not only improve the cyclic stability of Li–S batteries but also increase the sulfur loading and eventually maintain their energy density advantage.

With the significant progress made in the development of cathodes in Li–Sbatteries, the stability of the Li metal anode becomes a problem demanding a prompt solution in that Li reacts with most organic electrolyte solvents and Li salts instantly form a solid electrolyte interphase (SEI) layer on the Li surface [27]. Nevertheless, the SEI layer cannot withstand the mechanical deformation resulted from the Li-plating stripping process, attributing to its brittleness. Once a pinhole is formed in the SEI layer, the fresh Li metal will be exposed to the electrolyte, which will induce the growth of the treelike Li dendrite, and then such a substance will pierce through the separator and stimulate internal short circuits, leading to a significant safety hazard. Numerous studies have focused on the interface of Li metal and the electrolyte, by either strengthening the intrinsic SEI [27,28,29,30] or building a new interfacial layer [31,32], which suggests that Li dendrite growth originates mainly from the spatial inhomogeneity in charge distribution over the whole electrode surface. Uniform distribution of ionic flux needs to be achieved to prevent the Li dendrite’s growth from the origin. A flexible conductive matrix such as a carbon nanofiber was used to improve the mechanical properties of the SEI layer [33]. However, Cui et al. mentioned that the large surface area and high chemical reactivity of Li dendrites lead to a low coulombic efficiency [34]. Wu et al. also use an electrospun carbon nanofiber as anode material for long-term cycling in a lithium-ion battery [35]. It is therefore necessary to design an applicable structure of interlayer face to Li anode with both enhanced mechanical properties and high coulombic efficiency.

In this study, we present a novel modified Li–S cell structure with N-doped carbon nanofibers as the cathode interlayer and polyimides-based (PI-based) carbon nanofibers as the anode interlayer. The electrospun N-rich carbon nanofibers (MCNFs) interlayer was produced by using melamine (Mel) as one of the chemical reaction anhydride components during the PAA synthesis. MCNF functions as a current collector besides a polysulfide diffusion barrier. The PI-based CNF acted as an anode interlayer to ensure uniform charge distribution over the entire electrode surface. Both of the interlayers are carbon nanofibers without activation, which is cost-effective and environmentally friendly. Based on such a structure, this work aims to understand the behavior of electrodes with an interlayer during the charge–discharge cycles of Li–S batteries.

## 2. Results

### 2.1. Nanofiber Characterization

#### 2.1.1. Characterization of PI and PI+ Melamine

**Figure 1:**
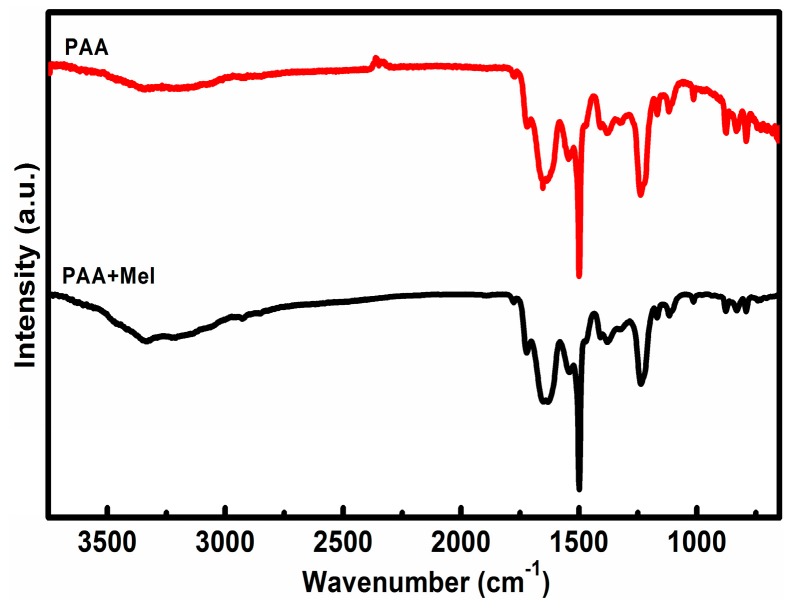
ATR-FTIR spectra of polyacrylic acid (PAA) and modified polyacrylic acid (MPAA).

The attenuated total reflectance Fourier Transform infrared spectroscopy (ATR-FTIR) spectrum exhibits a few characteristic bands of pyromellitic dianhydride/4,4′-oxydianiline (PMDA/ODA) polyimide as shown in Figure 1: 1722 cm^−1^ (symmetric C=O stretching, imide I), 1640 cm^−1^ (asymmetric C=O stretching, imide I), 1379 cm^−1^ (C–N–C stretching, imide II), 1500 cm^−1^ (C–C stretching of benzene ring), 1245 cm^−1^ (C–O–C stretching of ODA), 1652 cm^−1^ (C=O stretching, amide I) and 1553 cm^−1^ (C–N stretching, amide II) [36]. The band at 1489 cm^−1^ with modified polyacrylic imide (MPI) is related to the C=N stretching from melamine [37]. Peaks at 788–810 cm^−1^ corresponded to the absorption of benzene of ODA and PMDA groups. On the other hand, 3330 and 3225 cm^−1^ bands (N–H stretching) are totally absent, indicating that there are free –NH_2_ without reaction with –COOH in this case [38].

#### 2.1.2. Morphology of the Electrospun N-Rich Carbon Nanofibers

**Figure 2:**
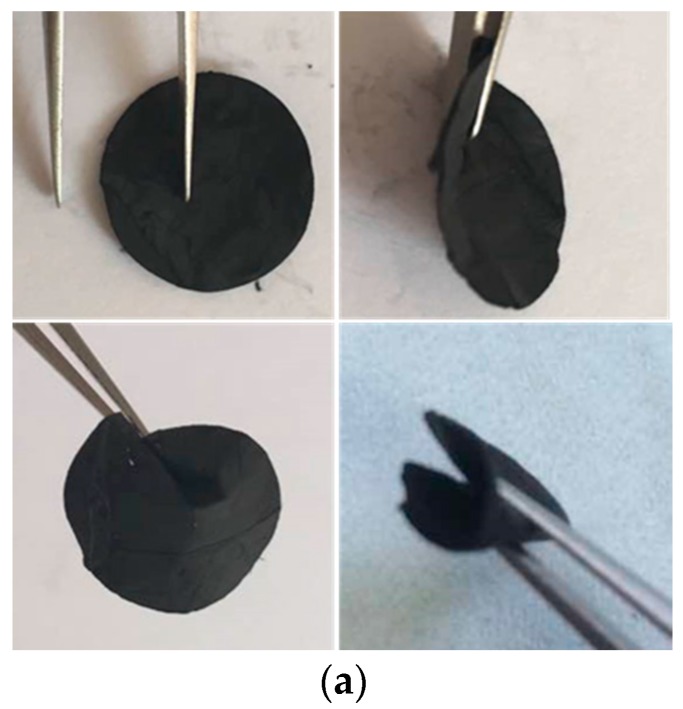

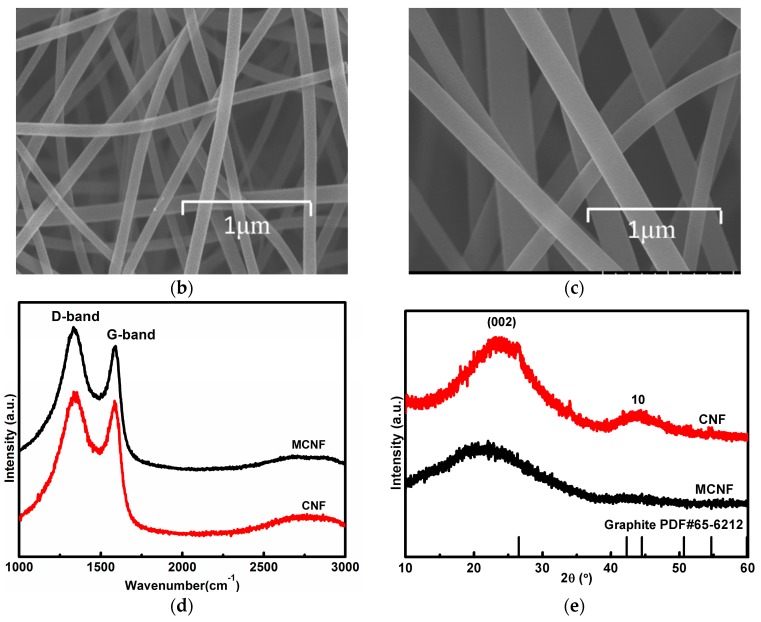
Digital image of N-rich carbon nanofibers (MCNF) (**a**) and SEM images of MCNF (**b**) and carbon nanofibers (CNF) (**c**); Raman spectrum of MCNF and CNF (**d**); X-Ray diffraction pattern of MCNF and CNF (**e**).

Figure 2a shows the digital image of MCNF, indicating high flexibility under a bending condition. Scanning electron microcopy (SEM) images of the MCNF after carbonization at 700 °C and CNF after carbonization at 900 °C are shown in Figure 2b,c, respectively. Both the MCNF and CNF are smooth and uniform. The fiber diameter is from 200 to 300 nm with CNF (Figure 2c) while the fiber diameter is from 80 to 100 nm with MCNF (Figure 2b). As reported [39], the fiber diameter is directly proportional to the concentration or viscosity of the electrospinning solution. To make the synthesized solution electrospinnable, more DMF was added when introducing melamine in the synthesis process. The concentration and viscosity of the MCNF electrospinning solution are smaller than those of the CNF electrospinning solution.

#### 2.1.3. Raman/X-ray Diffraction

The Raman spectra of MCNF and CNF show the appearance of three broad bands at 1332 (D), 1590 (G) and 2692 (2D) cm^−1^. Peaks at 1575 cm^−1^ (G band) and another at ~2880 cm^−1^ (2D band), historically designated as the G’ peak. The G band originates from the in-plane vibrations of aromatic carbon and is attributed to the doubly degenerate (E_2g_) phonon mode symmetry [36,37,38,39,40,41,42]. G’ peak is due to second-order zone boundary phonons, originating from a two-phonon double resonance process related to the band structure of the graphene layers. The D band initiates from vibrational of amorphous carbon. The integrated intensity ratio of D to G band (I_D_/I_G_) is ~2.8 with MCNF and ~2.3 with CNF, suggesting the formation of turbostratic structure.

The X-ray diffraction (XRD) patterns of MCNFs and CNF are shown in Figure 2e. The broad peaks of MCNF and CNF with around 24° and 44° can be indexed to the development of (002) and 10 diffraction lines, respectively (PDF Number: 65-6212) [43]. The width of band with MCNF is broader than that of CNF, which confirms that the ratio of turbostratic microstructure in MCNF is higher than in CNF.

#### 2.1.4. X-ray Photoelectron Spectroscopy Analysis

**Figure 3:**
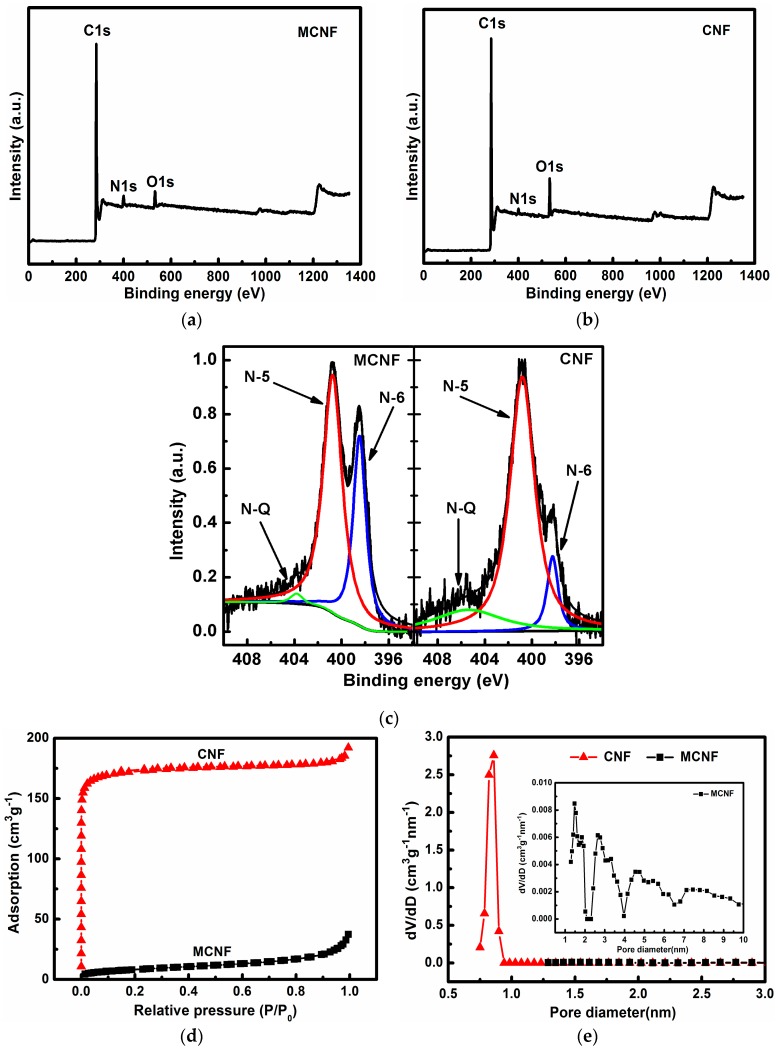
X-ray photoelectron spectroscopy spectra of (**a**) MCNF; (**b**) CNF; (**c**) N1s of CNF and MCNF; (**d**) N_2_ adsorption/desorption isotherms and (**e**) pore size distributions of samples.

X-ray photoelectron spectroscopy (XPS) was conducted to corroborate the nitrogen doping in the carbon lattice and to investigate the chemical status of elements in the MCNFs. As plotted in Figure 3a, the XPS survey scan spectra exhibits signals of C, O, and N elements. As expected, the content of nitrogen was increased by adding melamine as a component in the synthesis of PAA. The N atomic ratio of the MCNF is 5.11%, while the N atomic ratio of the CNF is 2.44%.

High-resolution XPS was performed to further investigate the bonding configurations of N atom in MCNF and CNF samples. Figure 3c shows the binding energy of N1s of the samples. As for both samples in Figure 3c, the complex N1s spectra can be further deconvoluted into three different component peaks at ~398.4 eV, 400.8 eV, and 403.8 eV, which can be assigned to the pyridinic (N-6), pyrrolic/pyridone (N-5) and quaternary (N-Q) nitrogen, respectively. According to previous studies, the N-Q nitrogen and N-6 nitrogen are both sp^2^ hybridized, and can improve the electronic conductivity of the carbon host, which is thus favorable for enhancing the electrochemical performance of carbon materials.

It was reported that N-doped graphene with pyrrolic and pyridinic N-dopants could bind LiPSs more strongly with larger binding energies (Eb) than pristine grapheme [44]. Except for graphitic N-dopants such as N-5, N-doped graphene with other N-dopants can provide stronger binding with soluble LiPSs. N-doped graphene with clustered pyridinic N-dopants binds soluble LiPSs much more strongly than electrolyte solvents do, indicating their effective role in anchoring the soluble LiPSs [45,46,47,48,49,50]. The total contents of N-Q and N-6 increase from 0.64% to 1.85% by introducing melamine into the matrix, as shown in Table 1, reflecting a stronger anchor effect with the soluble LiPSs.

Figure 3d,e illustrates the N_2_ adsorption and desorption isotherm of MCNF and CNF and Table S1 shows the elemental analysis of the MCNF after 100 cycles. Both of the samples exhibit the type I isotherm. The surface area of the CNF is 696 m^2^ g^−1^, which is mainly contributed by micropores with a diameter of 0.8 nm, as shown in Table 2. The pore volume is 0.29 cm^3^ g^−1^. The surface area of the MCNF is 30 m^2^ g^−1^ and pore volume is 0.05 cm^3^ g^−1^, indicating that the crosslinking network becomes denser through the introduction of melamine as one of the components, since the molecular weight of melamine is smaller than that of ODA [51,52,53].

### 2.2. Electrochemical Performance

Coin-type cells were assembled to evaluate the performance of different cell structures with interlayers. Both MCNF and CNF were tested as the anode interlayer for comparison. The coulombic efficiency decreases to 60% with MCNF after 200 cycles, as shown in Figure S1. It is assumed to be related to N doped in MCNF. Since the polar function group has a negative effect on coulombic efficiency, CNF was chosen as the anode interlayer for further testing.

**Figure 4:**
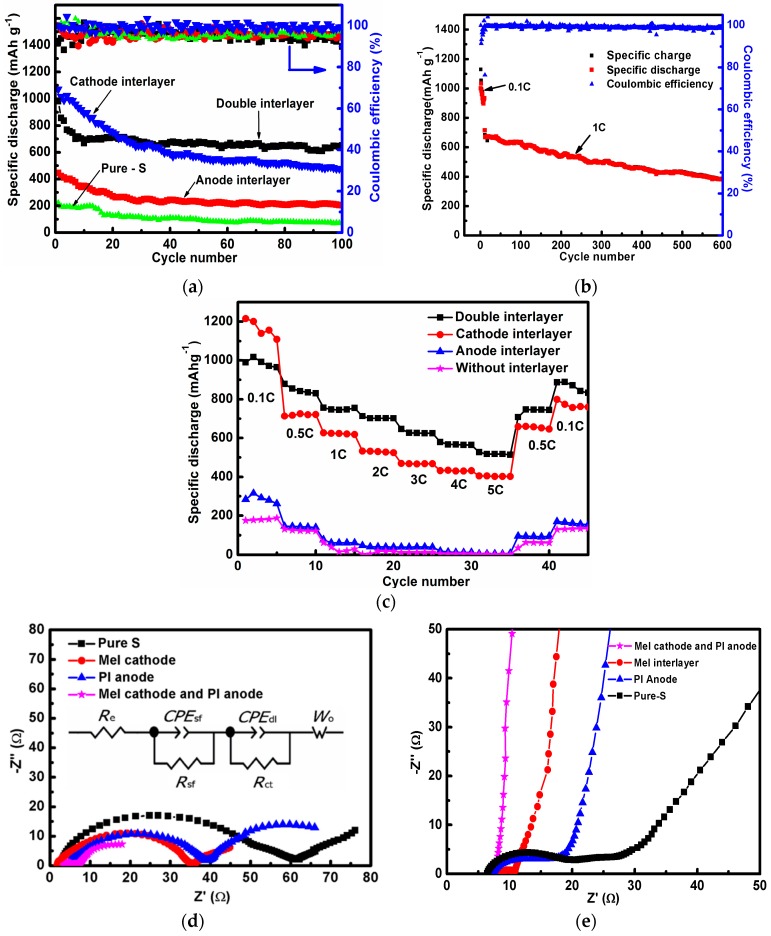
Cycling performance of the cells (**a**) at the rate of 0.1 C; (**b**) at the rate of 1 C, initial 10 cycles is 0.1 C the cycling performance, only for the cell with double interlayer; (**c**) the rate capabilities of the cells with different cell structures; electrochemical impedance spectra under the influence of an AC voltage of 0.1 mV; (**d**) the electrochemical impedance spectrum (EIS) before cycle with the equivalent circuit; electrolyte resistance (R_e_), constant phase angle element (CPE), interface impedance (R_sf_ // CEP_sf_), reaction impedance (R_dl_ // CEP_dl_), semi-infinite diffusion (W_o_) (**e**) the EIS after 100 cycles at the rate of 0.1 C.

Figure 4a shows the cycling performance of the cells with different structures at 0.1 C for 100 cycles. The discharge capacity of the first cycle remains at around 983 mAh g^−1^ with both the cathode interlayer and anode interlayer; and the discharge capacity remains at 1070 mAh g^−1^ with a cathode interlayer while the discharge capacity remains around 445 mAh g^−1^ with an anode interlayer. The discharge capacity is around 221 mAh g^−1^ without an interlayer. The reversible capacity remains at 651, 477, and 205 mAh g^−1^ after 100 cycles, with both cathode and anode interlayers, a cathode interlayer, and an anode interlayer, respectively (with a coulombic efficiency of about 94.4%, 98.3%, and 95.9%). In a sharp contrast, the cell without interlayer displays a serious capacity degradation during the cycling tests, and a very limited capacity, 76 mAh g^−1^, was preserved after 100 cycles. The drop ratio of the capacitance is the smallest with the double interlayer structure. The cell with anode interlayer has twice the specific capacity of a cell without an interlayer. After 100 cycles, the cell with double interlayer can still deliver a high capacity of 651 mAh g^−1^, which is nine times higher than that of the cell without interlayer. There is a drastic capacity loss during the initial cycles (1 to 20) for the double layer as compared to that of the single layer. The reason is that the PI-based CNF contains many micropores, which can adsorb polysulfides, and the large surface area requires many electrolytes and much energy to form SEI. The cycling process of a cell with both cathode and anode interlayers at 1C is presented in Figure 4b. The discharge capacity stays at 380 mAh g^−1^ after 600 cycles (with a coulombic efficiency of about 98.8%). It is assumed that the anode interlayer can help improve the capacity retention capability, while the cathode interlayer suppresses the polysulfide shuttling towards the anode.

Since the rate capability is considered as another essential parameter for batteries, the Li–S cells were also tested under various current densities from 0.1 to 5 C rate. Figure 4c is the rate performance at different current densities. For comparison, different cell structures were tested. First of all, the cell with double interlayers delivers an average discharge capacity of 987, 848, 751, 704, and 519 mAh g^−1^ at 0.1, 0.5, 1, 2, and 5 C, respectively, while the capacity returns to 887 mAh g^−1^ when the current density turns back to 0.1 C. In addition, the cell with cathode interlayer delivers an average discharge capacity of 1164, 720, 623, 529, and 404 mAh g^−1^ at 0.1, 0.5, 1, 2, and 5 C, respectively. The capacity returns to 799 mAh g^−1^, corresponding to the current density’s drop to 0.1 C. In addition, the cell with anode interlayer delivers an average discharge capacity of 286, 143, 65, 42, and 6 mAh g^−1^ at 0.1, 0.5, 1, 2, and 5 C, respectively. In the meantime, the capacity descends to 170 mAh g^−1^ when the current density turns back to 0.1 C. In the last place, the cell without interlayer delivered average specific capacities of 181, 124, 34, 13, and 2 mAh g^−1^ at current densities of 0.1, 0.5, 1, 2, and 5 C, respectively. Still, the capacity has a positive correlation with the current density, decreasing to 130 mAh g^−1^ when the latter turns back to 0.1 C.

Both the charge and discharge capacities of the Li–S cell with cathode interlayer are a lot higher than those without an interlayer. As the surface area of MCNF is constrained to as little as 30 m^2^ g^−1^, the polysulfide shuttling suppression is mainly contributed by the doped N atom, based on the XPS analysis. There is a relatively higher binding energy between the N atom and Li_2_S/polysulfides compared with that between –CH_3_ and Li_2_S/polysulfides, suppressing the diffusion rate of polysulfides and improving the active material utilization [43]. Both the charge and discharge capacities of the Li–S cell with anode interlayer are higher than those without an interlayer, especially after 20 cycles. Although the anode interlayer demonstrates lower capacities in high-power working conditions, the stability was quite remarkable, indicating that the anode interlayer can locally uniform the current density and prevent the Li dendrite growth.

A resistance comparison of the cathode interlayer, anode interlayer, double interlayers, and no interlayer is shown in Figure 4d, plotted by the electrochemical impedance spectrum. As can be seen, the charge transfer resistances of the cells with cathode interlayer, anode interlayer, double interlayers, and without an interlayer are 33.7, 34.8, 3.7, and 60.2 Ω, respectively. The smaller resistance contributes to fast electron transfer in cathode and better rate performance. The bottom illustrates that the cell with double interlayers provides electrically conducting pathways for improving the redox chemistry of sulfur, which not only facilitates fast charge collection/transport but also promotes a higher utilization of active materials. Elsewhere, the cell with cathode interlayer shows a smaller charge transfer process (R_ct_) value (33.7) than R_ct_ value (34.8) of the cell with anode interlayer. The resistance along with cycling was also investigated to explain the cycling stability. As is shown in Figure 4e, after 100 cycles, the resistance became quite stable, including a charge transfer resistance of less than 1.3, 2.2, 10, and 13.3 Ω with double interlayers, cathode interlayer, anode interlayer and without an interlayer, respectively. The small and stable resistance make for better cycling performance. The semicircle at high-frequency regions corresponds to interface impedance (a parallel connection of R_sf_ and CPE_sf_), which reflects the resistance to lithium ion diffusion through the contacting interface and Li_2_S/Li_2_S_2_ solid film [54,55,56]. The semicircle at the middle frequency region corresponds to charge transfer resistance (a parallel connection of R_ct_ and CPE_dl_) against the interfacial electrochemical reaction involved in charge transfer [57,58,59]. Before cycles, the R_sf_ of the cell with double interlayers is much lower than the others (especially compared to the cells with a single interlayer), which means that the double interlayers act as collectors (the R_sf_ of the cell with an anode interlayer is much lower than the cell without an interlayer). After cycles, the R_sf_ of the cell with a double interlayer decreases from 3.7 to 1.3 Ω, indicating improved interfacial wettability and lithium ion diffusion with cycling. The charge transfer resistance remains steady during cycling, indicating stable active sites for interfacial electrochemical reaction. It further reveals that the cell with double interlayers possesses good electrochemical kinetics, which reflects the good cycle performance of the cell.

**Figure 5:**
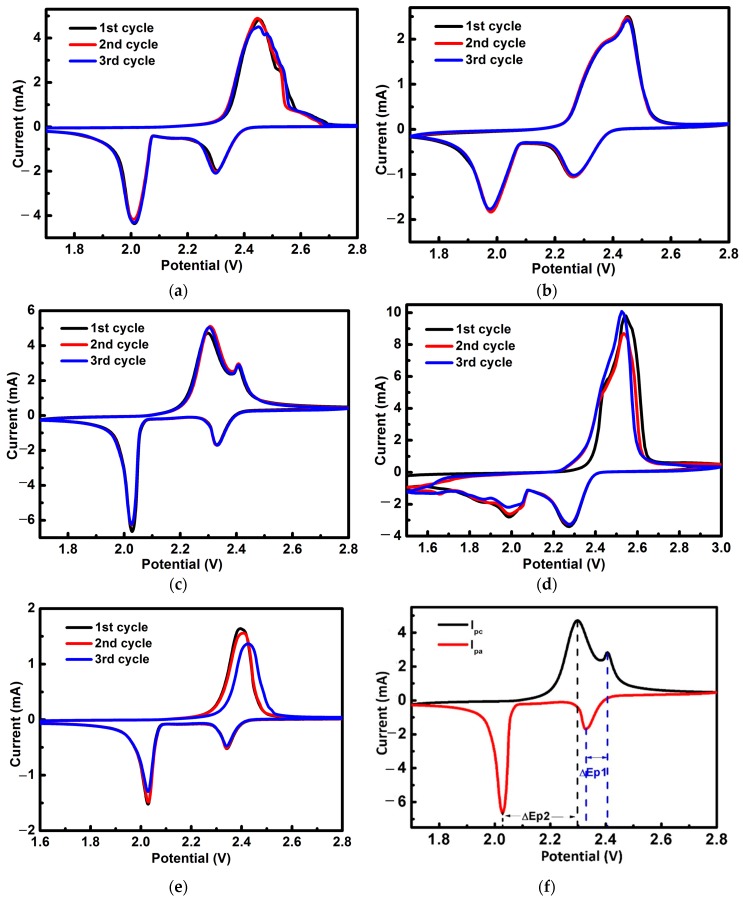
Cyclic voltammograms of the cell with a cathode interlayer (**a**); the cell with an anode interlayer (**b**) the cell with a double interlayer (**c**) the cell without an interlayer (**d**) and the cell with a double interlayer after 600 cycles at the rate of 1 C (**e**); the schematic of Δ*Ep*, Δ*Ep* (=*Epc* − *Epa*) (where *Ep* is peak potential, *Epc* is cathodic peak potential, *Epa* is anodic peak potential) (**f**).

To better understand the cycling performance, the cyclic voltammetry (CV) profiles were measured to identify the redox reactions for the cells with different cell structures (Figure 5). There are two distinct reduction peaks in the cathodic scan and two overlapping oxidation peaks in the anodic scan consistent with the discharge/charge curves, of which the four peaks are two pairs of redox peaks. The peak/plateau at ≈2.4 V is attributed to the reduction of elemental sulfur to higher-order polysulfides between S_8_ molecular and L_2_S_4_, while the peak/plateau at lower voltage (<2.1 V) is attributed to the further reduction of high-order polysulfides to low-order polysulfides between soluble Li_2_S_4_ and insoluble Li_2_S_2_ or Li_2_S [60].

In contrast to highly stable Li–S batteries achieved by adopting covalently-bonded sulfur polymers, which have extremely lower cathodic potential below 2.0 V, the interlayer has a limited effect on the peak positions. With a cathode interlayer, the cathodic potential is higher than the anode potential. The dominant cathodic peak is at 2.3 V with cathode interlayer and double interlayers, while it is 2.5 V with an anode interlayer and without an interlayer. The width of the redox spectra is narrower for the double interlayer cell. Also, the highly consistent overlap of the cathodic peaks indicates that the electrochemical process is highly stable with interlayers. The CV of the pure S electrode without an interlayer was also tested for comparison, as shown in Figure 5d, from which we can observe that the curve is not very stable.

If the redox process is reversible, the peak potential separation Δ*Ep* (=*Epc* − *Epa*) (where *Ep* is peak potential, *Epc* is cathodic peak potential, *Epa* is anodic peak potential) should be 59 mV at 25 °C for single electron transfer redox systems (Figure 5e). Table 3 shows that Δ*Ep* lies in the range from 80 mV to 480 mV, which indicates that the reactions of the cells with both the MCNF and CNF are quasi-reversible.

## 3. Discussion

### 3.1. The Role of Interlayer for Preventing Polysulfide Diffusion

**Figure 6:**
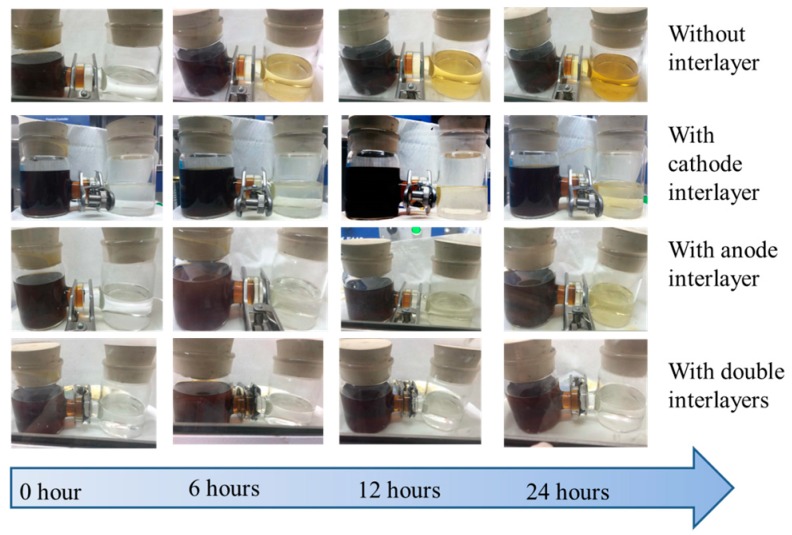
The optical images of the diffusion process of polysulfides of separator, cathode interlayer with separator, anode interlayer with separator, and cathode anode interlayers with separator in H type cell after variable resting times.

A polysulfides diffusion test was also conducted in an H-type electrolytic cell to monitor the effect of the interlayers in obstructing the shuttle of polysulfides. Figure 6 shows the color variation of the right section with different interlayers. The reddish-brown color of the section with two interlayers stayed at the same level over 24 h, indicating the great inhibition of polysulfides’ diffusion through the two interlayers. As is expected, the separator cannot suppress the diffusion of lithium polysulfides without interlayer. The polysulfides gradually diffused through the separator and reached the blank chamber as observed after 6 h. The same measurement was carried out with a separator with anode interlayer, cathode interlayer, and double interlayers. With an interlayer, the penetration time becomes longer, as shown in the pictures, to be concrete; the penetration time can be ranked as follows: without interlayer < anode interlayer < cathode interlayer < double interlayers. After 24 h, no obvious change of color in the right chamber can be observed with double interlayers, indicating that the double interlayer structure plays a vital role as a shield for polysulfides’ anions. Based on the comparison between anode and cathode interlayer performances, it can be concluded that the contribution of the cathode interlayer to shutting down the polysulfides is of more significance than that of the anode interlayer.

### 3.2. Cathode Interlayer

**Figure 7:**
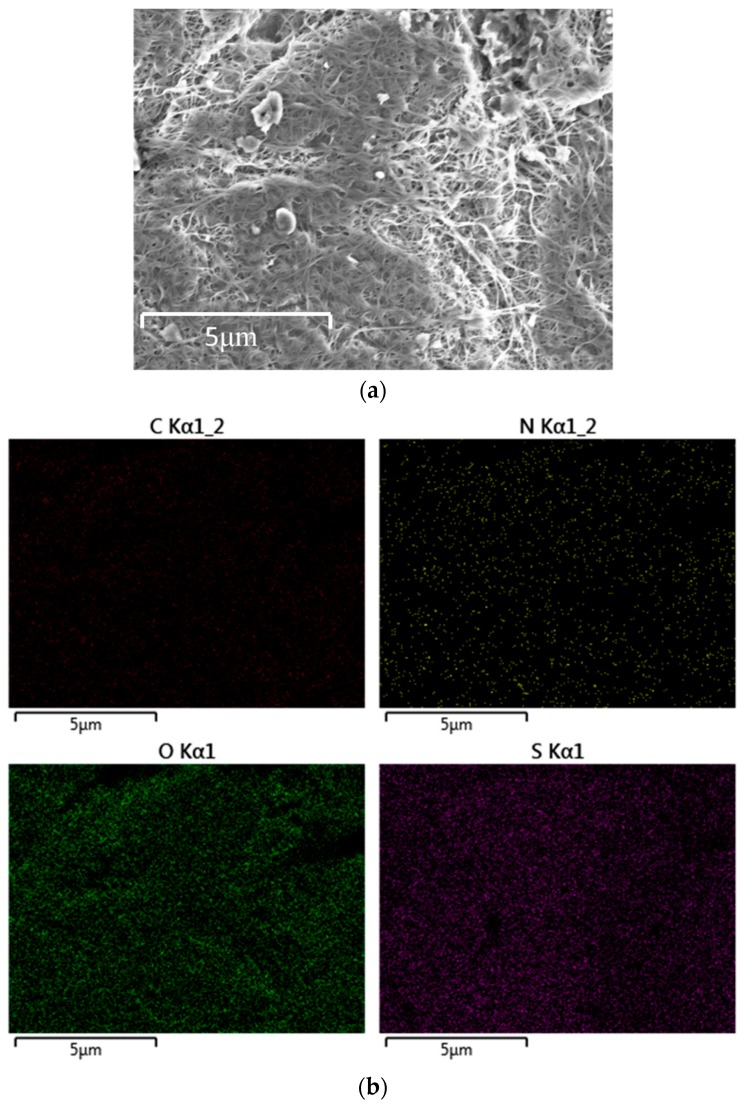
(**a**) SEM images of the interlayer after 100 cycles at 0.1 C; (**b**) the mapping of the cathode interlayer after 100 cycles at 0.1 C.

SEM images of the interlayer before and after 100 cycles at 0.1 C are shown in Figure 2c and Figure 7a, respectively, further explaining why the cells with the interlayer can considerably advance the performance of the Li–S cells. The polysulfides are evenly distributed on the MCNF rather than forming large agglomerates. Also, the energy dispersive spectrometer (EDS) results exhibited in Figure 7b further indicate that sulfur is distributed homogenously after cycles.

### 3.3. Anode Interlayer

**Figure 8:**
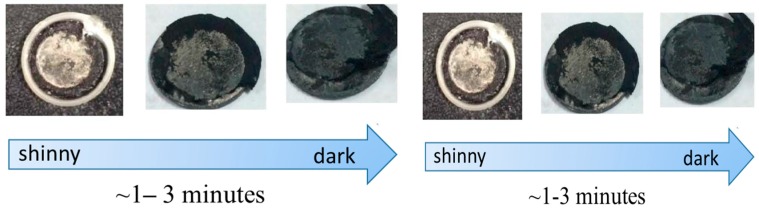
The picture of the surface belongs to the cell with an anode interlayer after 100 cycles at 0.1 C.

To understand the anode interlayer behavior, the cells were dissembled and a shiny interlayer surface was observed (Figure 8) after 100 cycles. The shiny surface faces the separator of the cell. After a few minutes the shiny surface became dark and dim.

**Figure 9:**
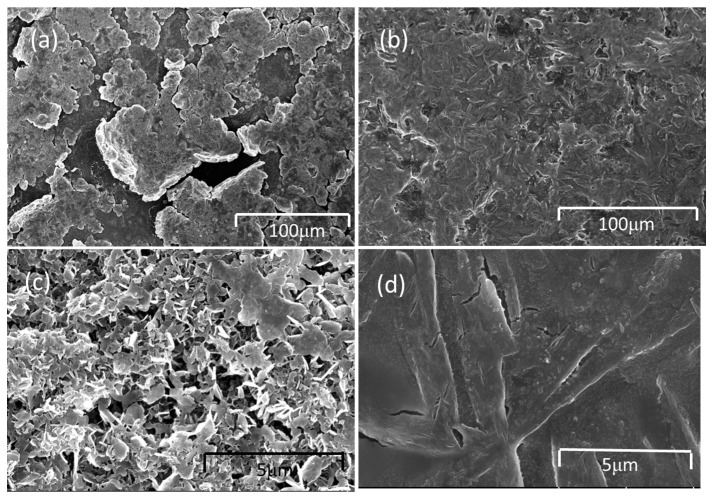

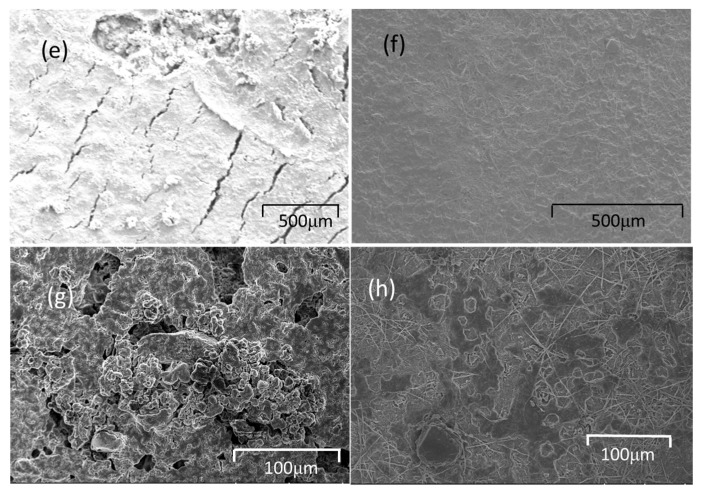
SEM images of the anode with and without interlayer after cycles at 0.1 C (**a**–**d**); the anode without interlayer after 100 cycles at 0.1 C (**a**,**c**); the anode with interlayer after 100 cycles at 0.1 C (**b**,**d**); SEM images of the anode with and without interlayer after cycles at 1 C (**e**–**h**); the anode without interlayer after 600 cycles at 1 C (**e**,**g**); the anode with interlayer after 600 cycles at 1 C (**f**,**h**).

The morphology of the surface belonging to the original Li electrode after 100 cycles at 0.1 C was checked under SEM, as shown in Figure 9a–d. Cracks from the original Li electrode were identified; the cracks are an order of magnitude smaller when using an anode interlayer, as shown in Figure 9a–d. With the increase in the number of cycles, the cracks became bigger and bigger, and shed from the Li electrode (Figure 9a,c). For the cell with an anode interlayer, the electrodeposited Li was compactly packed into the CNF layer, and the Li with CNF tightly adhered to the original Li electrode (Figure 9b,d). These images clearly confirm the formation of an embedded Li interlayer with cycling. Additionally, the surface of the interlayer electrode did not indicate any dendritic Li growth and the cracks are much smaller than the bare Li electrode.

The SEM images of the Li electrodes with the anode interlayer and without an interlayer after 600 cycles at 1 C are presented in Figure 9e–h. In the bare Li electrode, some large cracks were identified (Figure 9e,g). The cracks are more obvious in the cell cycle at the rate of 0.1 C. For the cell with an anode interlayer, the Li with CNF tightly adhered to the original Li electrode (Figure 9f,h). It can be seen that, the performance is the same at the low rate.

These results imply that CNF can present excellent mechanical robustness and withstand compressive stress, whereas bare Li without an interlayer could not afford to accommodate the excessive growth of the Li due to its inherent brittleness. Furthermore, the CNF interlayer slows down dendritic Li growth. A submicron-sized Li plate was found on the top layer of the interlayer. Although the surface area is as large as 696 m^2^ g^−1^ with CNF, the coulombic efficiency in the cell is high, as shown in Figure 5b. This should be related to the average pore size ~0.8 nm in the bulk of CNF, which is too small for Li crystals to penetrate.

The dramatic decrease of the charge transfer resistance with double interlayers can be contributed to the uniformly distributed S in MCNF facing the cathode and the thinner inactive porous layer for the redox reaction on the top of the anode.

## 4. Experimental

### 4.1. Materials

Sublimed sulfur (S) (98 wt %, Sinopharm Chemical Reagent Co., Ltd., Shanghai, China), Pyromellitic dianhydride (PMDA) (Mw = 218.12 g mol^−1^, Sinopharm Chemical Reagent Co., Ltd., Shanghai, China) lithium wafers (Li) (99.9%, Sinopharm Chemical Reagent Co., Ltd., Shanghai, China), *N*-methyl-2-pyrrolidone, NMP (99%, Sinopharm Chemical Reagent Co., Ltd., Shanghai, China), Poly(vinylidene fluoride) (PVDF) (ARKEMA, HSV900, Paris, France), *N*,*N*-dimethylformamide (DMF) (Xilong Chemical Co., Ltd., Shantou, China), Super P (from TIMCAL, Switzerland), Sinopharm Chemical Reagent Co., Ltd.), 4,4′-oxydianiline (ODA) (Mw = 200.24 g mol^−1^, Sinopharm Chemical Reagent Co., Ltd., Shanghai, China) and melamine Mel (99.0%, Sinopharm Chemical Reagent Co., Ltd., Shanghai, China) were used as received.

### 4.2. Fabrication of N-Drop Carbon Nanofibers

**Figure 10:**
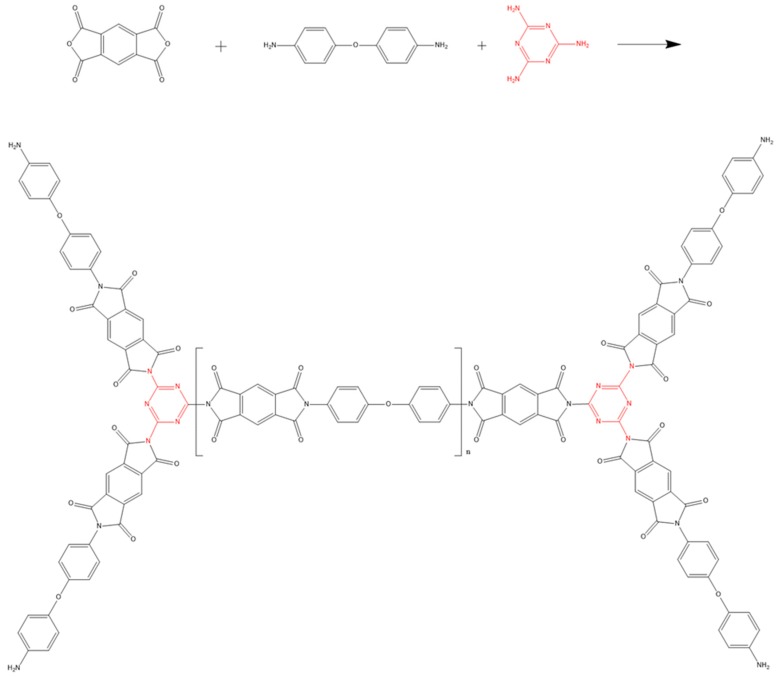
Scheme of the MPAA reaction.

To distinguish from our previous studies [43], melamine was introduced into the PAA synthesis process as an anhydride. The amide groups were not only from ODA in this case, but also from melamine, which is rich with nitrogen, as shown in Figure 10.

Next, 2.2 g PMDA, 2 g ODA, and 1.25 g melamine were dissolved in 30.0 g DMF to form a homogeneous polymer solution, which is used as an electrospinning solution. High-voltage electrospinning technology is used to prepare polymer nanofibers; by controlling the voltage during high-voltage electrospinning, the distance between the needle and the receiver and the viscosity of the precursor fluid can control the morphology of the polymer nanofibers [61,62,63,64,65,66,67,68,69].

During the electrospinning process, the polymer precursor was loaded into a syringe pump and a voltage of 23 kV was provided by a high-voltage power supply. A flow rate of 0.8 mL h^−1^ and a tip-to-collector distance of 20 cm were applied to ensure a stable electrospinning (Figure S2a). The as-electrospun nanofiber was collected as a flexible fabric, which was first increased from room temperature to 300 °C at a rate of 3 °C min^−1^ and maintained in an air flow for 30 min for imidization. Then the temperature was raised to 700 °C at the same heating rate and held for 1 h under an N_2_ atmosphere for carbonization. The carbon nanofibers constituted of melamine were labeled as MCNF, the weight of which was about 1.0 mg and was cut into wafers with a diameter of 16 mm to facilitate subsequent experiments. The MCNF was the cathode interlayer, and the anode interlayer was used as PI-based CNF, as in our previous studies [43]. The thickness of MCNF and PI-based CNF were both about 10 μm.

### 4.3. Characterization

SEM images were collected by a scanning electron microscope (LEO 1530, Oxford, Germany). XRD patterns were measured with D/MAX-RM 2000 (Rigaku, Tokyo, Japan) with Cu Kα radiation from 10° to 60° after the samples were sealed with Kapton tape (3M, Shanghai, China). Raman measurements (JobinYvon Labor Raman HR-800, Tokyo, Japan), were performed after the samples were sealed in a chamber with a glass window in the glove box. XPS data analysis was performed using Thermo Avantage software (Thermo Fisher Scientific, Cambridge, MA, USA) The surface chemical properties of the samples were characterized by XPS (Escalab 250, Cambridge, MA, USA). Elemental analysis (CHONS) was performed using FLASH EA1112 Elemental Analyzer (Thermo Fisher Scientific, Cambridge, MA, USA). The porosity of the cathode composite with open pores was measured using the Brunauere–Emmette–Teller (BET) method with nitrogen gas (Bellsorp-mini, BEL, Tokyo, Japan).

The sulfur cathode was fabricated by mixing 80 wt % of sulfur, 10 wt % of Super P, and 10 wt % of PVDF with NMP. The slurry was cast onto Al foil and dried at 55 °C in a vacuum oven for 24 h. Then the Al foil with slurry was cut into wafers with a diameter of 12 mm and the sulfur mass loading was about 4.5 mg cm^−2^. CR2032 coin cells were assembled in four kinds of structures in an argon-filled glove box with electrodes, separator (Celgard 2400, labeled as ST), and interlayers, including S|MCNF|ST|CNF|Li (Figure S2b); S|MCNF|ST|Li; S|ST|CNF|Li; and S|ST|Li. The electrolyte used in this study is 1.0 M lithium bis-trifluoromethanesulfonylimide (LiTFSI) in 1,3-dioxolane (DOL) and 1,2-dimethoxyethane (DME) (1:1 by volume), with 1 wt % LiNO_3_ additive.

The visible diffusion test was carried out in an H-type glass cell to examine the performance of the different interlayers. Four combinations of separators and interlayers, as mentioned above, were set in the H-type glass cell with 1.0 M LiTFSI in DOL and DME (1:1 by volume) with 1 wt % LiNO_3_ additive as the electrolyte. One side of the H-type glass cell with electrolytes was mixed with polysulfides. It is noticeable that the whole test was conducted in a glove box to isolate the influence of water and oxygen. The diffusion behavior of polysulfides across the separator or the interlayer was recorded by an optical camera.

Cyclic voltammetry (CV) and the electrochemical impedance spectroscopy (EIS) analysis were tested by an electrochemical workstation (CHI 600, Shanghai Chen Hua Instrument Company, Shanghai, China). The CV measurements were performed in the range of 1.5 to 3.0 V at a scanning rate of 0.1 mV s^−1^. The EIS spectra were measured at 5 mV amplitude with a frequency range from 0.1 Hz to 100 kHz. In this study, the potential is referred to the Li^+^/Li redox couple. Galvanostatic charge/discharge tests were carried out on a Land Battery Testing system (CT2001B, Wuhan LAND ekectronics Limited by Share, Wuhan, China). The current density set for the tests referred to the mass of sulfur in the cathode and varied from 0.1 C to 5 C.

## 5. Conclusions

In summary, a novel PI-based CNF with high nitrogen function groups contributed by melamine was used as a cathode interlayer and a PI-based CNF was applied as an anode interlayer to empower the electrochemical performance of an Li–S battery. MCNF, as a pseudo-upper current collector, not only reduces the charge transfer resistance of sulfur cathodes significantly, but also captures the migrating active material at the end of cycles. Also, the PI-based CNF served as an anode interlayer to uniform charge distribution over the entire electrode surface. This approach offers the possibility to improve the cycling stability at high S loading. The electrochemical analysis shows that the cell with double interlayers delivers an initial discharge capacity of 983 mAh g^−1^. After 100 cycles, the discharge capacity was 651 mAh g^−1^ and the coulombic efficiency was 95.4% at 0.1 C. What is more, the discharge capacity was kept at 380 mAh g^−1^ after 600 cycles with a coulombic efficiency of about 98.8% at 1 C. The cell configuration provides good electronic contact between the carbon matrix and the active material, and improves the utilization of sulfur for further improvements in cycling stability. The extended utilization of the Li deposits within the CNF matrix can help reduce polarizations. A double interlayer structure is the proper method to overcome the shuttle phenomenon and ensure the cycling stability of Li–S cells. Taking advantage of the facile fabrication process and excellent performance, the novel configuration proposed would be of interest for scalable production and fabrication for high-energy Li–S batteries.

## Figures and Tables

**Table 1:** The elemental analysis of CNF and MCNF.

	CNF	MCNF
C1s Atomic %	90.12%	90.42%
N1s Atomic %	2.44%	5.11%
O1s Atomic %	7.45%	4.58%
Pyridinic (N-6)	0.23% (398.4 eV)	1.76% (398.4 eV)
pyrrolic/pyridine (N-5)	1.76% (400.8 eV)	3.25% (400.8 eV)
quaternary (N-Q)	0.41% (405.4 eV)	0.09% (403.8 eV)

**Table 2:** The surface area and pore volume of CNF and MCNF.

Sample	Surface Area (m^2^ g^−1^)	Pore Volume (cm^3^ g^−1^)
CNF	696	0.29
MCNF	30	0.05

**Table 3:** The ∆*Ep* of different structure cells.

Structure	Double Interlayer	Cathode Interlayer	Anode Interlayer	Without Interlayer
∆*Ep*1 (mV)	80	170	190	280
∆*Ep*2 (mV)	280	440	390	480

## Supplementary Materials

The following are available online at www.mdpi.com/1996-1944/10/4/376/s1, Figure S1: Cycling performance of the cells with double MCNF interlayer at the rate of 0.1 C. Figure S2: A schematic diagram of electrospinning (a) and the double interlayer cell (b). Table S1: The elemental analysis of the MCNF after 100 cycles.
