# Supplementary material for: Effects of Electrospun Carbon Nanofibers’ Interlayers on High-Performance Lithium–Sulfur Batteries
Source: Materials (Basel). 2017 Mar 31;10(4):376. doi: 10.3390/ma10040376 (PMC5506899; doi:10.3390/ma10040376)
Supplement: Supplementary file 1 [file materials-10-00376-s001.pdf]

Effects of electrospun carbon nanofibers interlayers on high performance Lithium Sulfur batteries

Tianji Gao[3], TrungHieu Le[2], Ying Yang[1, 2] *, Zhihao Yu[1], Zhenghong Huang[4], Feiyu Kang[4]

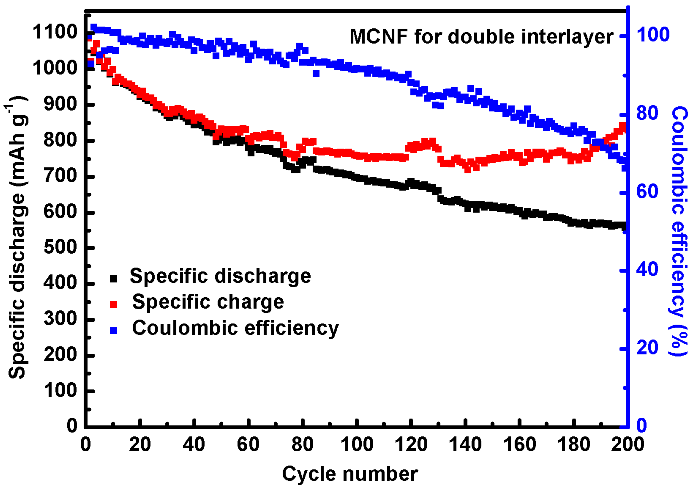

Figure S1. Cycling performance of the cells with double MCNF interlayer at the rate of 0.1 C.

Table S1. The elemental analysis of the MCNF after 100 cycles.

Name	Atomic %	Title
Li1s	26.27	Li1s Scan
C1s	28.21	C1s Scan
N1s	3.44	N1s Scan
O1s	42.08	O1s Scan

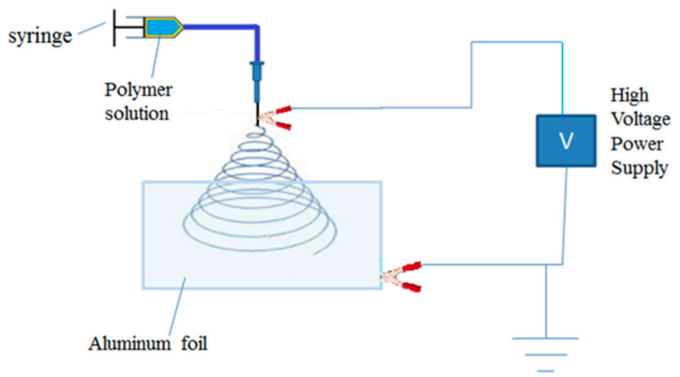

(a)

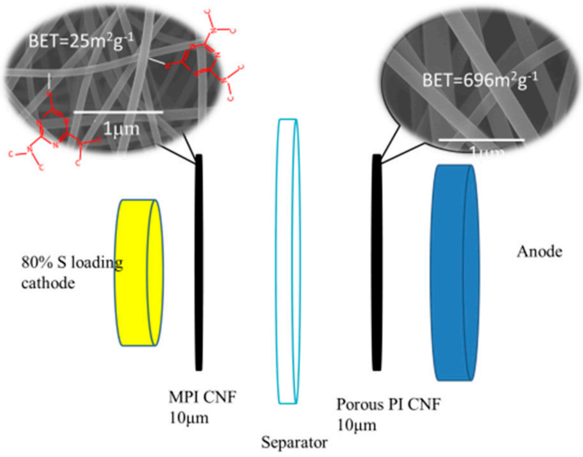

(b)

Figure S2. A schematic diagram of electrospinning (**a**) and the double interlayer cell (**b**).
